# Effects of *Coix* seed extract, *Lactobacillus paracasei* K56, and their combination on the glycolipid metabolism in obese mice

**DOI:** 10.1111/1750-3841.16474

**Published:** 2023-01-30

**Authors:** Wei Zhang, Xiuzhen Jia, Yuhan Xu, Qiaoling Xie, Meizhen Zhu, Zifu Zhao, Jingyu Hao, Haoqiu Li, Jinrui Du, Yan Liu, Wei‐Hsien Liu, Xia Ma, Weilian Hung, Haotian Feng, Hongwei Li

**Affiliations:** ^1^ School of Public Health Xiamen University Xiamen China; ^2^ Inner Mongolia Dairy Technology Research Institute Co. Ltd. Hohhot China; ^3^ Yili Innovation Center Inner Mongolia Yili Industrial Group Co., Ltd. Hohhot China

**Keywords:** Coix seed extract, glycolipid metabolism, inflammation, *Lactobacillus paracasei* K56, obesity, respiratory exchange ratio

## Abstract

**Abstract:**

*Coix* seed extract (CSE) and probiotics have been reported to regulate glycolipid metabolism through different modes of action. We tested the effects of CSE, *Lactobacillus paracasei* K56, and their combination to determine whether they have synergistic effects on glycolipid metabolism of obese mice. We fed male C57BL/6J mice with high‐fat diet for 8 weeks to establish an obesity model. The obesity mice were selected and divided into five groups: the model control group and four intervention groups. After 10 weeks of continuous gavage intervention, the mice in the intervention groups exhibited lower body weight (lower about 2.31–4.41 g, vs. HFD 42.25 g, *p* < 0.01), and epididymal (lower about 0.58–0.92 g, vs. HFD 2.50 g, *p* < 0.01) and perirenal fat content (lower about 0.24–0.42 g, vs. HFD 0.88 g, *p* < 0.05); decreased fasting blood glucose, total cholesterol, triglycerides, and VLDL; and increased HLDL, respiratory exchange ratio, energy expenditure, and amount of exercise performed. K56 + CSE‐combined intervention groups were more effective in lowering blood glucose, IL‐1β, and TNF‐α levels than the CSE and K56 alone interventions. The content of fatty acid synthase and SREBP‐1c protein in liver tissue was lower. The combination has synergistic effects on weight control, fat reduction, and blood glucose regulation by improving the chronic inflammatory state and reducing the content of lipid synthesis‐related enzymes of obese mice, which can hinder chronic disease progression.

**Practical Application:**

*Coix* seed extract can be used in obese people to regulate abnormal glucose and lipid metabolism and delay the development of chronic diseases.

## 1 INTRODUCTION

Obesity and obesity‐related metabolic diseases have become growing global public health problems. According to the World Health Organization, overweight and obesity cause 2.8 million deaths and 35.8 million disability‐adjusted life years (DALYs) each year, accounting for approximately 2.3% DALYs; furthermore 1/5 of adults worldwide will become obese (World Health Organization, 2017). Over the past decade, the prevalence of overweight and abdominal obesity in the Chinese population has increased rapidly, accounting for nearly 45% of the global obese population (NCD Risk Factor Collaboration (NCD‐RisC), 2016). Moreover, the “Report on Nutrition and Chronic Diseases of Chinese Residents (2020)” shows that more than half of the Chinese population is obese or overweight. Therefore, the incidence and mortality of chronic diseases are expected to escalate (Yuejiao, 2020).

*Coix lacryma‐jobi* L. var. *ma‐yuen* (Roman.) Stapf is a plant species belonging to the Poaceae family, whose dried and mature seed kernels are edible and have medicinal properties (Li et al., 2020). The main active components of *Coix* seeds are esters, unsaturated fatty acids, carbohydrates, and lactams (Bi et al., 2019; Peng et al., 2020). Recent pharmacological studies have shown that the polysaccharides, proteins, fatty acids, and polyphenols of *Coix* seeds have pharmacological effects such as regulating glucose and lipid metabolism and intestinal microbiota (Bi et al., 2019; Li et al., 2020; Peng et al., 2020).

Probiotics can also improve gut dysbiosis and are beneficial in preventing or treating obesity (Kootte et al., 2012; Xiao et al., 2014). *Lactobacillus casei*, *Lactobacillus paracasei*, and *Lactobacillus rhamnosus* are the most widely studied and applied *Lactobacillus* probiotics and are commonly used as food supplements in dairy products (Hill et al., 2018). Both animal experiments and human trials have revealed that *Lactobacillus* spp. prompts weight loss and improves glucose and lipid metabolism (Brahe et al., 2015; Han‐ying et al., 2021; Kanazawa et al., 2021; Park et al., 2013; Wang et al., 2020).

Both Coix seed extract (CSE) and probiotics can regulate glycolipid metabolism and intestinal microbiota; however, their modes of action differ. CSE regulates glycolipid metabolism through the absorption of its functional components, whereas probiotics directly regulate disturbances in the intestinal microbiota. Therefore, the combined use of CSE and probiotics may have synergistic effects. In this study, we determined the effects of CSE, *L. paracasei* K56, and their combination on weight loss, reduction in lipid levels, and regulation of blood glucose levels in high‐fat diet‐induced obese mice. We also explored their potential mechanisms behind these effects.

## 2 MATERIALS AND METHODS

### 2.1 Source of materials

CSE and *L. paracasei* K56 bacteria powder (K56) were provided by Beijing Yili Technology Development Co., Ltd (Beijing, China), and the recommended dose for humans was 1 mg/d (Jia‐qi et al., 2022). Preparation of CSE: The plant material was *Coix lacryma‐jobi* L. var. *ma‐yuen* (Roman.), using water and ethanol extraction, and the extraction ratio is 1:10. The procedure was that 1.2 kg dry powder and ground CSE were extracted with 95% ethanol reflux for 2 h, the extracts were combined in a rotary evaporator and concentrated, then the extract was dried in a vacuum drying oven at 80°C to obtain 102.3 g of CSE (Peng et al., 2022), which was frozen at −4°C. The active ingredients are Coix seed fat, protein, carbohydrates, and so on. The CSE used in this experiment contains >30% polysaccharides. For *L. paracasei* K56, 10^11^ colony‐forming units were obtained from Shenghe Biotechnology Co., Ltd (Taiwan, China), and the recommended dose for humans was $1\times 10^{7}\;\mathrm{cfu}/d$. The recipe groups are shown in Table 1.

**TABLE 1:** Animal experimental protocol

Group	Test substance	Recommended dose for humans (mg/d or cfu/d)	Intervention dose in mice (mg/d kg or cfu/d kg)	Gavage concentration (mg/ml or cfu/ml)
ND	Normal saline	–	–	–
HFD	Normal saline	–	–	–
Group 1	CSE	1.00	166.67	16.67
Group 2	K56	10^7^	1.67 × 10^6^	1.67 × 10^5^
Group 3	CSE	0.50	83.33	8.33
	K56	10^6^	1.67 × 10^5^	1.67 × 10^4^
Group 4	CSE	1.00	166.67	16.67
	K56	10^6^	1.67 × 10^5^	1.67 × 10^4^

Abbreviation: CSE, *Coix* seed extract.

*p* < 0.05, compared with ND.

*p* < 0.05, compared with HFD.

### 2.2 Ethical approval statement

All experimental procedures were performed in accordance with the guidelines of the Institutional Animal Care and Use Committee of the Laboratory Animal Center of Xiamen University and the International Association of Veterinary Editors guidelines for the Care and Use of Laboratory Animals. Protocols for animal use were reviewed and approved by the Animal Ethical and Welfare Committee of the Laboratory Animal Center of Xiamen University (Approval No. XMULAC20200185).

### 2.3 Animal experiments

According to our previous experiences, the success rate of high‐fat feeding obesity model was 40% (Han‐ying et al., 2021), and the samples of obesity mice required for this experience (Mera et al., 1998), the C57BL/6J male mice (*n* = 150) with an average body weight of 19.02 ± 0.88 g, were purchased from the Shanghai SLAC Laboratory Animal Co., Ltd. The mice were reared at 22°C, 10%−60% humidity, 12/12 h light–dark cycle, and free access to water. After 1 week of adaptive feeding, 12 mice were selected by the random number method and continued to be fed with normal diet for 8 weeks as the ND group, in which the mice were fed normal chow (Beijing Keao Xieli Feed Co., Ltd.; Beijing Feed Certificate (2018) 0673) for 8 weeks. The remaining mice were fed a high‐fat diet (Research Diets, Inc, D12492), in which fat accounted for 60% of the heat energy, for 8 weeks to establish mouse models of obesity. High‐fat feed was purchased from Jiangsu Shuangshi Laboratory Animal Feed Department [Su Feed Certificate (2017) 05005]. Mice displaying a 40% increase in body weight after feeding on high fat were considered successful obesity models and were randomly divided into 1 model control (HFD) and 4 intervention groups, with 12 mice in each group. The sample calculation was performed according to Mera et al. (1998) who consider the effect of treatment between the experimental groups on the outcome variables of interest.

The CSE and K56 doses used in the intervention groups are listed in Table 1. In the CSE and K56 alone intervention groups, the mice were administered CSE and K56 doses that were 10 times the recommended human doses according to guidance on Human and Animal Dose Conversion (State Administration for Market Regulation (2020)), and this guidance was based on the approximate range of human and mouse body surface area conversion. In the ND and HFD groups, the mice were administered normal saline via gavage during the 10‐week intervention period. The gavage volume of all groups was set at 0.1 ml/10 g mouse body weight. During the intervention period, the ND group was fed with normal chow (Beijing Keao Xieli Feed Co., Ltd.; Beijing Feed Certificate (2018) 0673), and the other groups were fed with a high‐fat diet (Research Diets, Inc, D12492), in which fat accounted for 60% of the heat energy. During the 10‐week intervention period, the body weight and feed intake per cage were measured weekly, and the energy intake was calculated based on the food intake. Fasting blood glucose (FBG) level was measured at weeks 0, 4, 8, and 10. At the end of the intervention, eight mice in each group were randomly selected to undergo oral glucose tolerance tests (OGTT). After a 12‐h fasting, the mice were immediately given a 20% glucose solution by gavage according to their body weight (10 µl/g). Blood was drawn from the tail vein of the mice at 0, 30, 60, and 120 min after glucose administration to measure the blood glucose concentration, using a blood glucose meter (Sannuo Biological Sensing Co., Ltd.).

### 2.4 Monitoring of animal metabolism

At the end of the intervention, four mice were randomly selected from each group to be monitored by the TSE PhenoMaster (12‐channel) system for 4 days, which measured the respiratory entropy metabolism and voluntary movement of mice. Each mouse was caged independently and could eat and drink ad libitum. The ND group was fed a normal diet, whereas the other groups were fed a high‐fat diet. The first 48 h were considered an adaptation period, and the data obtained were discarded. Thereafter, the stable metabolic monitoring started at 2:00 p.m. and ended at 2:00 p.m. the next day; a data point was recorded every 5 min for each mouse.

The respiratory exchange ratio (RER) and energy expenditure (EE_1_) were calculated based on the O_2_ consumption and CO_2_ production measured on the exhaust gas of each cage. The body weight was used to correct EE_1_ to obtain EE_2_, and the lean body mass was used to correct EE_2_ to obtain EE_3_.

### 2.5 Tissue sampling and index testing

At the end of the intervention, the mice were made to fast for 12 h. Their eyeballs were removed for blood collection after inhalation anesthesia with 4% isoflurane (Shenzhen Reward Life Technology Co., Ltd), and then the mice were killed by rapid vertebral dislocation. The body fat (peritesticular and perirenal fat) and liver tissues were rapidly collected, rinsed with normal saline, drained, and weighed. The blood was centrifuged at 2000 rpm (382 *g*) and 4°C for 15 min, and the supernatant was collected to measure biochemical indicators. All samples were frozen at −80°C for later use.

#### 2.5.1 Lipid body ratio

$$\begin{array}{ccc} & & Lipid\;body\;ratio\;\left(\%\right)=\left[(Peritesticular\;fat\;mass\;\left(g\right)\right.\\ & & +\left.Perirenal\;fat\;mass\;\left(g\right))/Body\;mass\;\left(g\right)\right]\times 100\% \end{array}$$

(Li et al., 2019).

#### 2.5.2 Blood lipids

Serum was collected using an automatic biochemical analyzer (Mindray BS‐220) and supporting kits to detect TC, TG, HLDL, and VLDL.

#### 2.5.3 Serum insulin (INS), leptin (LEP), adiponectin (ADP), uncoupling protein 1 (UCP1), IL‐1β, and TNF‐α

The serum was taken to measure the INS, LEP, ADP, UCP1, IL‐1β, and TNF‐α levels of mice with mouse enzyme‐linked immunosorbent assay kit (ELISA), according to the manufacturer's instructions (Shanghai Sanyan Biotechnology Center). The insulin resistance index (HOMA‐IR) and islet β‐cell function (HOMA‐β) were calculated according to the following formula:

$$HOMA-IR\;=\frac{INS\;\left(\mathrm{mUI}/l\right)\times \;FPG\left(\mathrm{mmol}/l\right)}{22.51}$$

$$HOMA-\beta \;=\frac{20\times INS\;\left(\mathrm{mUI}/l\right)}{PFG\;\left(\mathrm{mmol}/l\right)-3.5}$$

#### 2.5.4 Hepatic glucose– and lipid metabolism‐related factors

Liver tissue was homogenized by adding an appropriate amount of normal saline, centrifuged at 3000 r/min for 10 min, and the supernatant was taken to measure the expression of *uncoupling protein 2* (UCP2), lipoprotein lipase (LPL), fatty acid synthase (FAS), sterol‐regulatory element‐binding transcription factor (SREBP‐1c), and cholesterol 7α‐hydroxylase (CYP7A1) protein in liver tissue by ELISA, according to the manufacturer's instructions (Shanghai Sanyan Biotechnology Center).

#### 2.5.5 Hematoxylin–eosin (H&E) staining of liver tissue

The live liver tissue samples were fixed using 4% paraformaldehyde for more than 24 h. The dehydrated paraffin embedding process is as follows: 75% alcohol 4h–85% alcohol 2h–90% alcohol 2h–95% alcohol 1h–anhydrous ethanol I 30min–anhydrous ethanol II 30min–alcohol benzene 5–10min–xylene I 5–10min–xylene II 5–10min–wax I 1h–wax II 1h–wax III 1 h. Then, it was cut into paraffin sections with a thickness of 4 µm with a microtome (RM2016, Shanghai Leica Instruments Co., Ltd) for hematoxylin–eosin (H&E) staining. The following process was Harris hematoxylin for 3–8min—distilled water—stained with eosin dye for 1–3min–distilled water–95% alcohol I 5min–95% alcohol II 5min–absolute ethanol I5min–absolute ethanol 5min–xylene I5min–xylene 5min. Finally, live tissue sections were analyzed by microscopy (Leica‐DM4 B, Germany).

### 2.6 Statistical analysis

The data are expressed as the mean, median, standard deviation, and percentiles. Repeated measures data were analyzed for variance using multivariate variance. If the data distribution is normal and the variance is homogeneous, one‐way analysis of variance (one‐way ANOVA) was used to compare the data between groups of one‐way measurement indicators, and the LSD method was used for pairwise comparisons between groups. If the data distribution does not satisfy the normal distribution, the nonparametric test Kruskal–Wallis *H* was used for comparison between groups, and the Nemenyi method was used for pairwise comparison of the overall mean between groups. Hypothesis test level *a* = 0.05.

## 3 RESULTS

### 3.1 Feed intake and body weight

At the beginning of the intervention (week 0), the average body weights of the mice in the HFD group and ND groups were significantly different, being 32.82 ± 1.53 g and 26.45 ± 1.94 g, respectively (*p* < 0.001). Therefore, high‐fat diet‐induced obesity mice model was successfully established. No significant difference in body weight was observed among the obese mice groups (*p* > 0.05), and the regrouping was reasonable. The average total feed intake and energy intake per mouse were not significantly different between the HFD and the intervention groups (*p* > 0.05, Figure 1).

**FIGURE 1:**
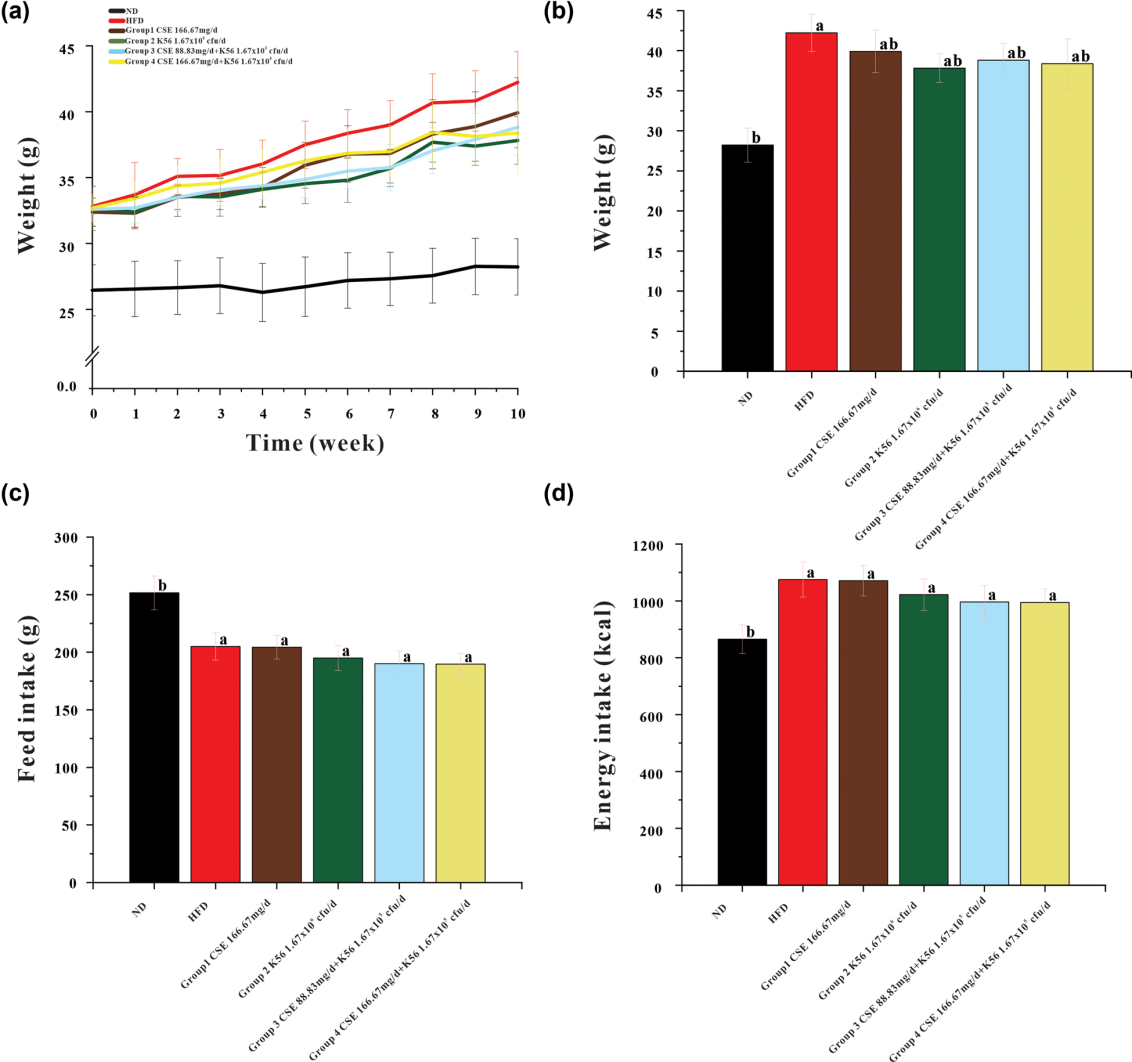
Results of body weight and feed intake: (a) body weight during intervention; (b) end‐of‐intervention body weight in each group; (c) total feed intake; (d) total energy intake. Data are means ± SD (*n* = 12); a: *p* < 0.05, compared with ND, and b: *p* < 0.05, compared with HFD.

A repeated‐measure test was performed using the body weight values of the mice obtained during the 10‐week intervention. The results showed that both the CSE and K56 administration and intervention time influenced mice weight in each group. As shown in Figure 1, the weight gain of the HFD group was higher than that of the intervention groups, and at the end of week 6, the difference in weight between each intervention group and the HFD group was statistically significant (*p* < 0.05).

At the end of the intervention, the average body weight of each intervention group was lower than that of the HFD group (*p* < 0.05), indicating that all interventions could effectively control the weight gain of high‐fat diet–induced obese mice. In the CSE + K56 intervention group, the K56 dose was 1/10 of the dose of the K56 intervention group, and the weight gain was slightly higher than that in the K56 intervention group (*p* > 0.05). The combined intervention presented slightly lower weight gain than the CSE intervention (*p* > 0.05), regardless of whether the CSE dose was lower than or equal to that in the CSE intervention.

### 3.2 Fat distribution

The fat weight and lipid body ratio of the mice in each intervention group were lower than those of the HFD group (*p* < 0.05, Figure 2A‐C). In the H&E section of the liver tissue, it was found that a large number of fat vacuoles formed in the liver tissue of the HFD group, indicating that high‐fat feeding increased not only perirenal and peritesticular fat, but also fatty liver occurrence. After the intervention of the test substance, the fat vacuoles in the liver became smaller and less, which was similar to the liver tissue structure of the ND group (Figure 2D). These findings indicated that all interventions could reduce the visceral fat (liver, perirenal, and peritesticular) accumulation caused by a high‐fat diet in mice. In the CSE + K56 intervention group, the K56 dose was 1/10 of the dose of the K56 intervention group, and the fat weight was slightly higher than that in the K56 intervention group (*p* > 0.05); the fat weight was also slightly higher compared to CSE alone (*p* > 0.05), regardless of whether the composition of CSE dose was lower than or equal to CSE alone.

**FIGURE 2:**
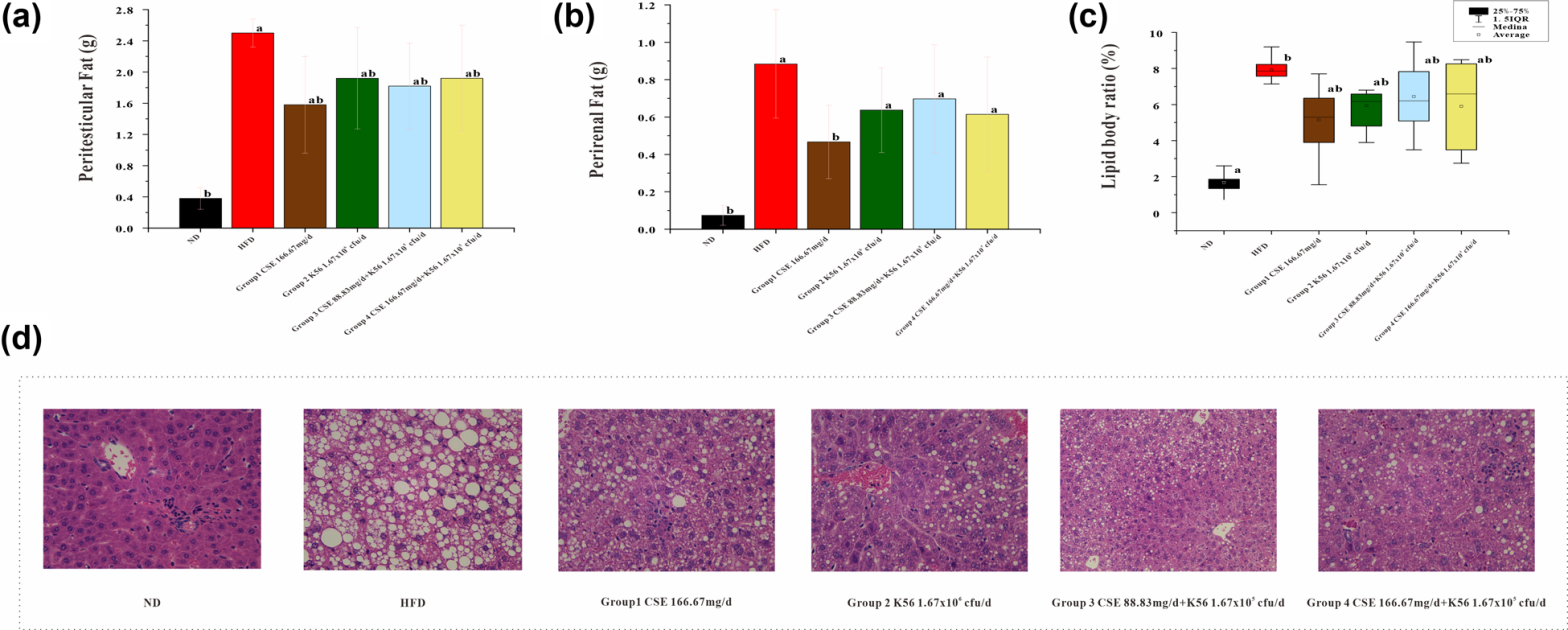
Results of fat weight: (a) peritesticular fat weight; (b) perirenal fat weight; (c) lipid body ratio; and (d) liver hematoxylin–eosin (H&E). Data are means ± SD (*n* = 12); a: *p* < 0.05, compared with ND, and b: *p* < 0.05, compared with HFD.

### 3.3 Blood lipids levels

High‐fat diet increased the serum levels of TC, TG, VLDL and decreased HLDL (Figure 3), Subsequently, all interventions decreased the serum levels of TG, TC, and VLDL and increased HLDL levels in obese mice. In each intervention group, the TC, VLDL, and HLDL levels approached the levels of the ND group (*p* > 0.05). No significant differences were observed in the levels of blood lipids among the intervention groups (*p* > 0.05), regardless of their differences in CSE and K56 doses.

**FIGURE 3:**
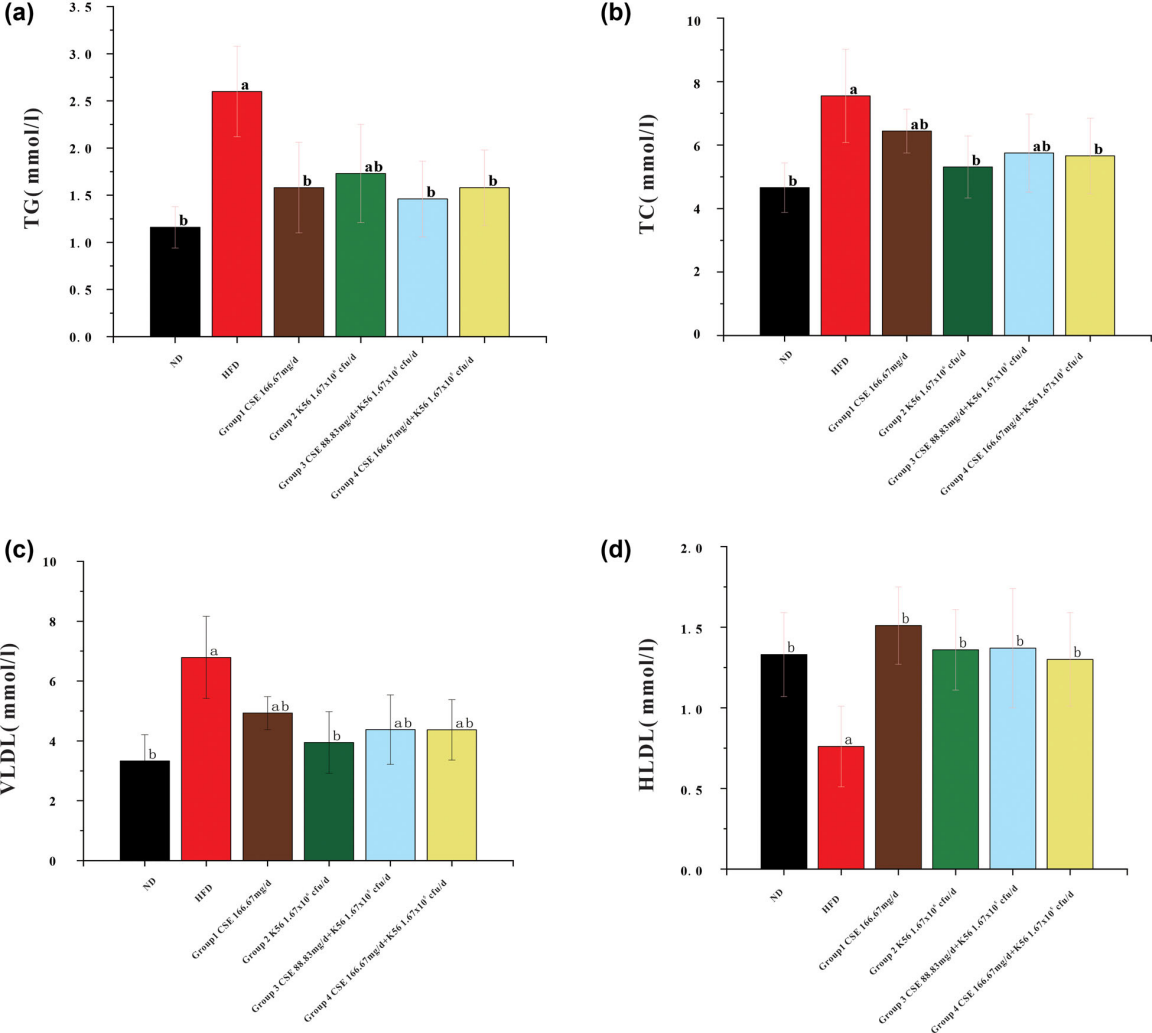
Results of blood lipids levels: (a) TG (triglycerides); (b) TC (total cholesterol); (c) VLDL (very low‐density lipoprotein); (d) HLDL (high‐density lipoprotein). Data are means ± SD (*n* = 12); a: *p* < 0.05, compared with ND, and b: *p* < 0.05, compared with HFD.

### 3.4 FBG level

As opposed to the HFD group, each intervention group showed a downward trend in the FBG levels (Figure 4). At week 4, the FBG levels of each intervention group were lower than those of the HFD group (*p* < 0.05), indicating that all interventions could effectively control the increase in FBG level caused by the high‐fat diet. In addition, the FBG levels of the obese mice in the CSE + K56 group were lower than those in the CSE group at week 4 (*p* < 0.05), indicating that the combined intervention of CSE and K56 could quickly control FBG levels. At the end of the intervention, obese mice were gavaged with CSE alone; they exhibited the best regulation of FBG levels.

**FIGURE 4:**
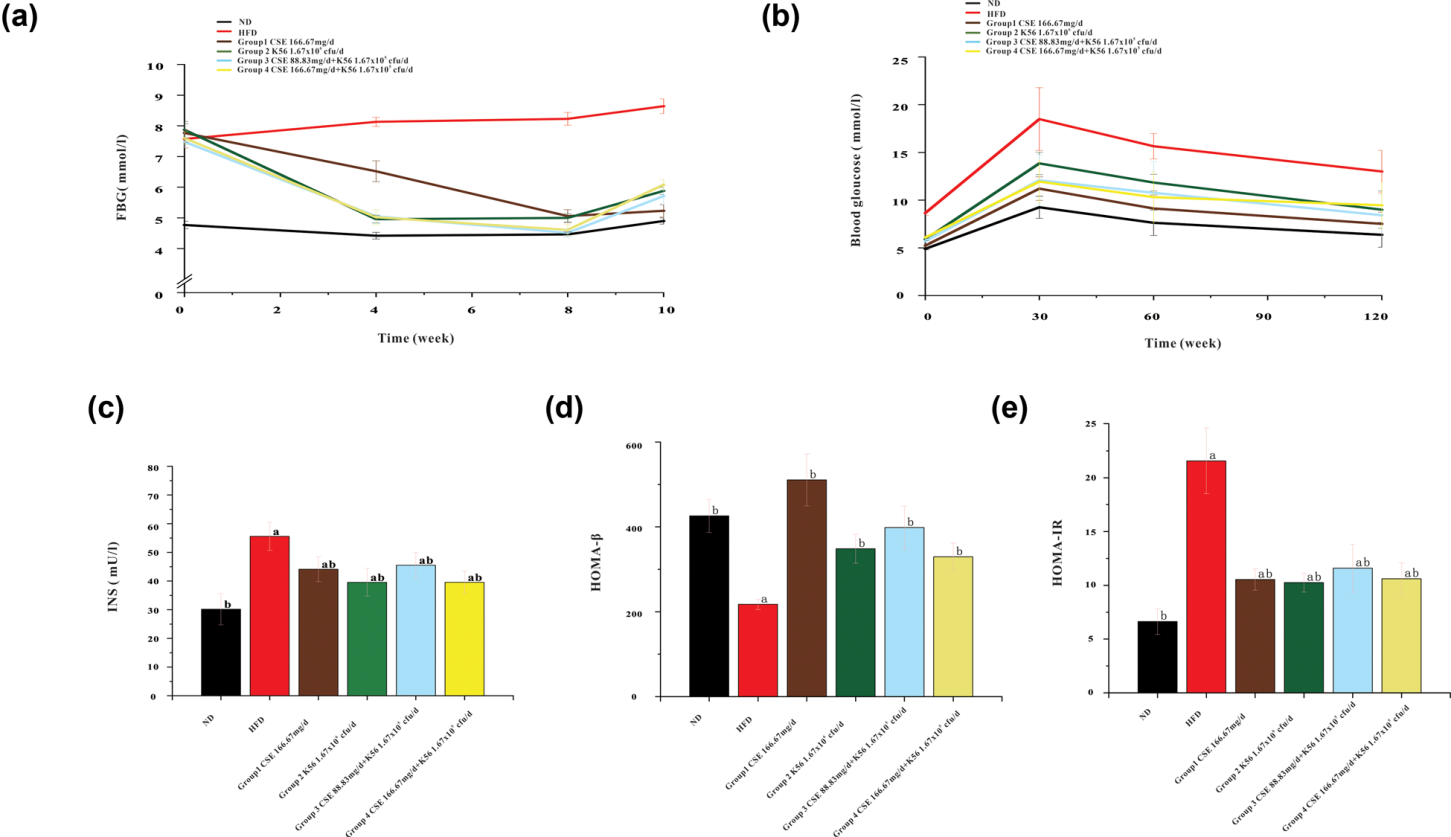
Results of markers of glucose metabolism: (a) fasting blood glucose; (b) OGTT (oral glucose tolerance tests); (c) serum INS (insulin) level; (d) HOMA‐IR (insulin resistance index); and (e) HOMA‐β (insulin β‐cell function). Data are means ± SD (*n* = 12); a: *p* < 0.05, compared with ND, and b: *p* < 0.05, compared with HFD.

### 3.5 OGTT test

The area under the curve (AUC) of each intervention group was smaller than that of the HFD group, indicating that all interventions enhanced the regulation of blood glucose in obese mice (Figure 4). The AUC of the CSE + K56 intervention group was lower than that of the K56 intervention group, suggesting that the combined intervention improved the blood glucose regulation ability of obese mice to a greater extent than K56 alone. Among the intervention groups, obese mice treated with CSE alone exhibited the highest ability to regulate blood glucose level.

### 3.6 Serum INS, HOMA‐IR, and HOMA‐β levels

In each intervention group, the INS and HOMA‐IR levels were lower and the HOMA‐β levels were higher than those of the HFD group (*p* < 0.05, Figure 4). The high‐fat diet damaged the islet cell function of the obese mice; thus, the mice in the HFD group might have developed insulin resistance. However, all interventions reduced the islet function damage in the high‐fat diet‐induced obese mice, with the function being similar to that of the ND group. The islet function of the mice that underwent the CSE + K56 intervention was not significantly different from that of the mice that were treated with K56 alone (*p* > 0.05). Likewise, the INS and HOMA‐IR levels were similar (*p* > 0.05) in the combined and CSE alone interventions, regardless of the CSE dose. Although HOMA‐β levels were slightly higher (*p* < 0.05) in the combined intervention than in the CSE intervention, their islet function was comparable.

### 3.7 Indicators related to energy metabolism

RER values were calculated by dividing the volume of CO_2_ produced by the volume of consumed O_2_. When different substrates are oxidized in the body, they have corresponding RERs, which can be used to determine the energy consumption substrates of the body. Studies have shown that when the body completely consumes only carbohydrates as the substrate, the RER is equal to 1; when the body does not completely consume glucose as substrate and also consumes fat or protein as the substrate, the oxygen demand increases and the RER values decrease. When the only substrate consumed is fat, RER is approximately 0.70 (Shuwen, 2018; Wanjing & Zhao, 2018; Xiaofeng, 2019).

The RER values of the HFD group remained stable over time at approximately 0.76, whereas the RER values of the ND group had a clear circadian rhythm, and the RER was 0.93−1.00 (Figure 5), suggesting that the energy consumption substrate of high‐fat modeled obese mice mainly comes from fat, and glucose is less used as an energy consumption substrate. After the intervention of the test substance, the RER values of the obese mice increased, indicating that the test substance intervention reduced the proportion of fat in the substrate consumed by the obese mice and increased the proportion of glucose in the metabolic substrate. Compared with the single use of K56, the dose of K56 in the composition is 1/10 of the single use, but the RER of the composition is not significantly different from that used alone (*p* > 0.05), and the effect is similar; compared with the single use of CSE, no matter the composition of CSE dose was lower than or equal to single use, but the RER of the composition was higher (*p* < 0.05). The results showed that the combined use of CSE and K56 has a synergistic effect, which can increase the proportion of glucose consumption as the metabolic substrate.

**FIGURE 5:**
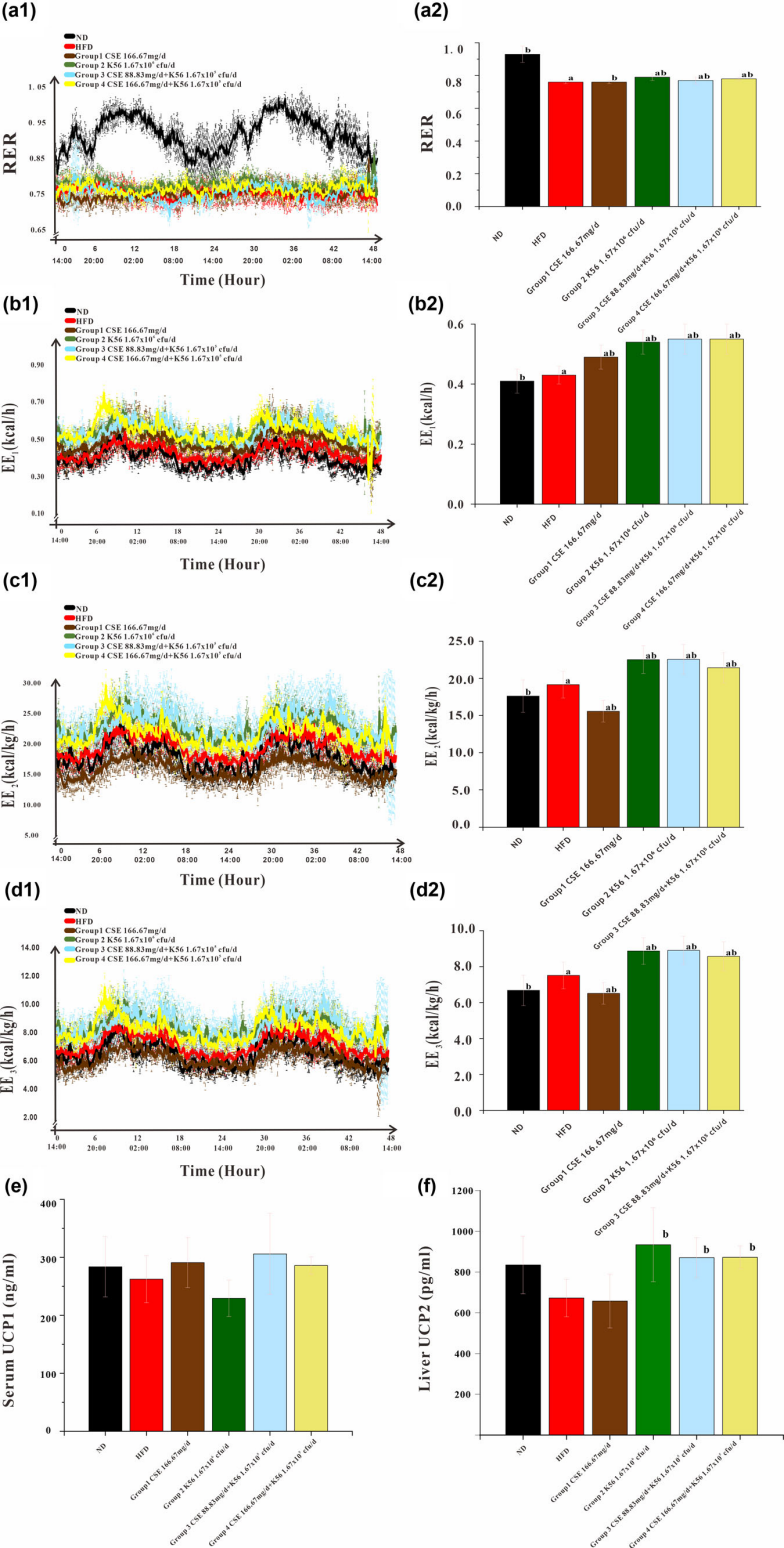
Energy metabolism‐related indicators: (a1) change in the respiratory exchange ratio (RER) over time; (a2) comparison of the mean RER of each group; (b1) change in the energy expenditure (EE_1_) over time; (b2) comparison of the mean EE_1_ of each group; (c1) change in the energy expenditure (EE_2_) over time; (c2) comparison of the mean EE_2_ of each group; (d1) change in the energy expenditure (EE_3_) over time; (d2) comparison of the mean EE_3_ of each group; (e) serum UCP1 (uncoupling protein 1); and (f) liver UCP2 (uncoupling protein 2). The body weight was used to correct EE_1_ to obtain EE_2_, and the lean body mass was used to correct EE_2_ to obtain EE_3_. Data are means ± SD (*a1‐d2, n=4; e‐f, n* = 12); a: *p* < 0.05, compared with ND, and b: *p* < 0.05, compared with HFD.

The EE of the body is mainly composed of three parts, including resting metabolic rate, thermic effect of food, and activity energy expenditure (Xiaofeng, 2019). As shown in Table 2, the EE_1_ of the HFD group was higher than that of the ND group, but the amount of exercise was lower than that of the ND group, indicating that the total energy consumption was related to body weight. Although the activity levels of obese mice were lower than those of normal mice, the total energy expenditure of obese mice was higher than that of the ND mice. After the interventions, the EE_1_ and amount of exercise of obese mice were higher than those of the HFD group. UCP1 and UCP2 belong to the family of mitochondrial transport proteins that uncouple the transport of protons across the inner mitochondrial membrane from electron transport and ADP synthesis of ATP, thereby generating heat (Boss et al., 2000; Kim et al., 2022). UCP1 diverts energy from ATP synthesis to thermogenesis in the mitochondria of brown adipose tissue by catalyzing a regulated leak of protons across the inner membrane (Echtay et al., 2002). However, the results of this experiment showed that UCP1 in the intervention group was not higher than that in the HFD group (*p* > 0.05), indicating that the intervention of the test substance may not increase body heat production through UCP1. The role of UCP2 is to protect organisms from free radicals (Diano & Horvath, 2012; Echtay et al., 2002), and the lower UCP2 would lower resting energy expenditure (Ahmadi et al., 2020). The hepatic UCP2 content of the intervention group was higher than that of the HFD group (*p* < 0.05), except for Group 1 (CSE 166.67 mg/d) (*p* > 0.05), indicating that the intervention of the test substance K56 or K56 + CES can improve in generating heat (Figure 5), reducing free radicals, lowering inflammation levels (Figure 6), and reducing fat accumulation (Figure 2D). These findings showed that the interventions increased the exercise volume and energy consumption of obese mice.

**TABLE 2:** Result of energy metabolism‐related indicators

Group	Test substance	Dose (mg/d kg or cfu/d kg)	RER ($\bar{X}\pm S$)	EE_1_ ($\bar{X}\pm S$, kcal/h)	EE_2_ ($\bar{X}\pm S$, kcal/kg/h)	EE_3_ ($\bar{X}\pm S$, kcal/kg/h)	Amount of exercise (m/d)
ND	Normal saline	–	0.93 ± 0.05^b^	0.41 ± 0.04^b^	17.62 ± 2.18^b^	6.86 ± 0.85^b^	1299.19 ± 308.01^b^
HFD	Normal saline	–	0.76 ± 0.01^a^	0.43 ± 0.03^a^	19.16 ± 1.79^a^	7.51 ± 0.74^a^	713.00 ± 133.28^a^
Group 1	CSE	166.67	0.76 ± 0.01^a^	0.49 ± 0.04^a^, ^b^	15.57 ± 1.45^a^, ^b^	6.51 ± 0.60^a^, ^b^	1180.00 ± 146.19^b^
Group 2	K56	1.67 × 10^6^	0.79 ± 0.02^a^, ^b^	0.54 ± 0.04^a^, ^b^	22.53 ± 1.87^a^, ^b^	8.87 ± 0.74^a^, ^b^	1352.57 ± 277.44^b^
Group 3	CSE + K56	83.33 + 1.67 × 10^5^	0.77 ± 0.02^a^, ^b^	0.55 ± 0.05^a^, ^b^	22.57 ± 2.00^a^, ^b^	8.90 ± 0.79^a^, ^b^	1186.29 ± 160.69^b^
Group 4	CSE + K56	166.67 + 1.67 × 10^5^	0.78 ± 0.01^a^, ^b^	0.55 ± 0.05^a^, ^b^	21.44 ± 2.03^a^, ^b^	8.57 ± 0.81^a^, ^b^	1464.86 ± 272.45^b^

Abbreviations: CSE, *Coix* seed extract; RER, respiratory exchange ratio.

^a^ *p* < 0.05, compared with ND.

^b^ *p* < 0.05, compared with HFD.

**FIGURE 6:**
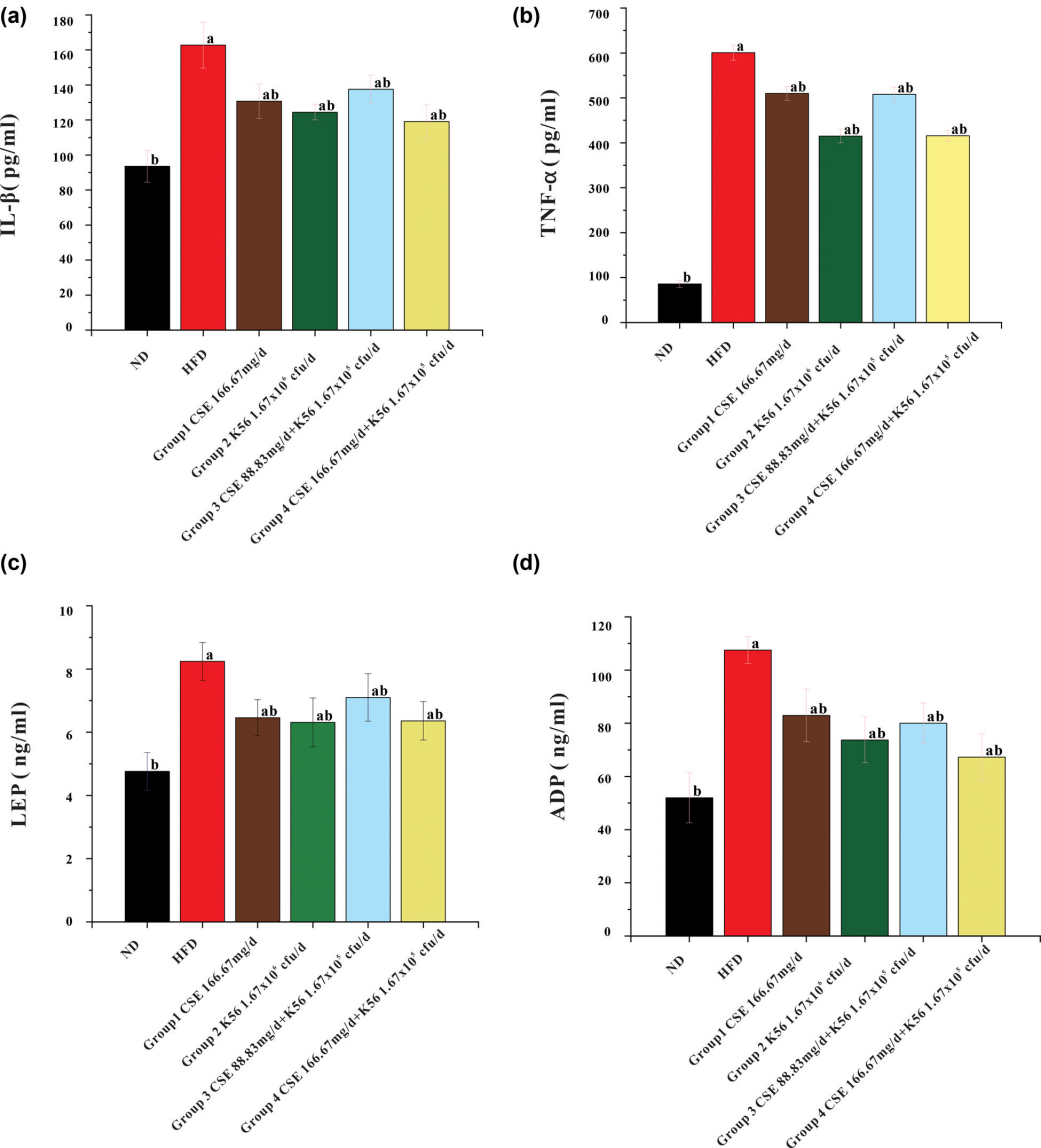
Results of serum glycolipid metabolism‐related indicators: (a) inflammatory factors IL‐1β; (b) inflammatory factors TNF‐α; (c) serum LEP (leptin) level; and (d) serum ADP (adiponectin) level. Data are means ± SD (*n* = 12); a: *p* < 0.05, compared with ND, and b: *p* < 0.05, compared with HFD.

After adjusting body weight and lean body mass content, only the mice in Group 1 (CSE 166.67 mg/d) consumed less energy (EE_2_ and EE_3_) than the HFD group. According to the body weight and body fat data, Group 1 (CSE 166.67 mg/d) displayed the worst weight control effect and lowest fat content among the intervention groups. Therefore, the EE_1_ of Group 1 (CSE 166.67 mg/d) was higher than that of the HFD group, which may be mainly affected by body weight and lean body mass. The dose of K56 in the CSE + K56 intervention group was 1/10 of the K56 intervention group, but the EE_1_, EE_2_, and EE_3_ of the CSE + K56 intervention group were not significantly different from K56 intervention group (*p* > 0.05), and the effect is similar; no matter the dose of CSE in the CSE + K56 intervention group was lower than or equal to the CSE intervention group, the EE_1_, EE_2_, and EE_3_ of the CSE + K56 intervention were higher (*p* < 0.05). These results showed that the combined use of CSE and K56 had a synergistic effect on increasing the energy metabolism of obese mice.

### 3.8 Serum inflammatory factors IL‐1β and TNF‐α

IL‐1β is produced by activated macrophages and is the initiator of inflammatory response, which can trigger a series of cascade reactions at the cellular and subcellular levels, inducing the production of various inflammatory factors such as IL‐6 (Dinarello & van der Meer, 2013). TNF‐α is produced by mononuclear macrophages and regulates multiple inflammatory and autoimmune processes (Uysal et al., 1997). Previous studies have shown that obese individuals have elevated levels of IL‐1β and TNF‐α, which can induce insulin resistance, promote free fatty acid (FFA) production, and affect glucose and lipid metabolism (Bastard et al., 2006; Bouter et al., 2010; Uysal et al., 1997).

As shown in Figure 6, the serum levels of IL‐1β and TNF‐α in each intervention group were lower than those in the HFD group (*p* < 0.05). IL‐1β serum levels in Group 4 (CSE 166.67 mg/d + K56 1.67 × 10^5^ cfu/d) were lower than those in the other intervention groups (*p* < 0.05). TNF‐α serum level in Group 4 (CSE 166.67 mg/d + K56 1.67 × 10^5^ cfu/d) was lower than Group 1 (CSE 166.67 mg/d) (*p* < 0.05). Therefore, all interventions reduced the serum levels of IL‐1β and TNF‐α, and the combined intervention was more effective in reducing inflammation than the CSE and K56 alone interventions.

### 3.9 Serum LEP and ADP levels

LEP is a protein hormone secreted by adipose tissue, which can regulate feeding behavior, inhibit fat synthesis, and promote lipid decomposition (Münzberg & Morrison, 2015). ADP is a polypeptide or protein secreted by adipocytes, which inhibits hepatic glucose production, enhances fatty acid oxidation in skeletal muscle, and has beneficial metabolic effects that jointly promote energy homeostasis in the body (Wang & Scherer, 2016). Studies showed that elevated LEP levels in obese individuals lead to leptin resistance, a risk factor for obesity (Myers et al., 2008). Meanwhile, obesity‐induced insulin resistance can lead to downregulation of ADP receptors in muscle and liver (Kadowaki et al., 2006).

As shown in Figure 6, the serum levels of LEP and ADP in each intervention group were lower than those in the HFD group (*p* < 0.05). ADP serum levels of Group 4 (CSE 166.67 mg/d + K56 1.67 × 10^5^ cfu/d) were lower than those in the other intervention groups (*p* < 0.05). All interventions could reduce serum LEP levels, relieve leptin resistance, inhibit fat synthesis, promote lipid decomposition, relieve insulin resistance, restore ADP receptor function, inhibit hepatic glucose production, and lower blood glucose. Compared with the single use of CSE and K56, the serum ADP of CSE and K56 used in combination intervention was lower.

### 3.10 LPL, CYP7A1, FAS, and SREBP‐1c levels in liver tissue

LPL regulates key enzymes involved in lipid metabolism, mediates FFA oxidation, and provides energy to body tissues by hydrolyzing triglyceride‐rich lipoproteins in the plasma (Basu & Goldberg, 2020). CYP7A1 is a liver‐specific microsomal cytochrome P450 enzyme system that catalyzes the rate‐limiting step in the decomposition of cholesterol into bile acids; thus, it plays a role in maintaining the bile acid–cholesterol balance and lipid homeostasis in the body (Pikuleva, 2008). FAS is a key enzyme in the fatty acid regeneration process, as it catalyzes the synthesis of palmitic acid from acetyl‐CoA and malonyl‐CoA (16:0) (Jensen‐Urstad & Semenkovich, 2012). SREBP‐1c is a transcription factor that regulates the expression of enzymes that synthesize endogenous cholesterol, fatty acids, triglycerides, and phospholipids (Foufelle & Ferré, 2002; Griffin & Sul, 2004).

As shown in Figure 7, mice in the intervention groups presented lower levels of LPL, CYP7A1, FAS, and SREBP‐1c in the liver tissue than those of mice in the HFD group (*p* < 0.05). FAS levels in Group 4 (CSE 166.67 mg/d + K56 1.67 × 10^5^ cfu/d) were lower than those in the other intervention groups (*p* < 0.05). The SREBP‐1c content in Group 4 (CSE 166.67 mg/d + K56 1.67×10^5^ cfu/d) was lower than that in Group 1 (CSE 166.67 mg/d) (*p* < 0.05). The CSE + K56 intervention reduced the SREBP‐1c and FAS content in the liver tissue to a greater extent than that of the CSE and K56 alone interventions.

**FIGURE 7:**
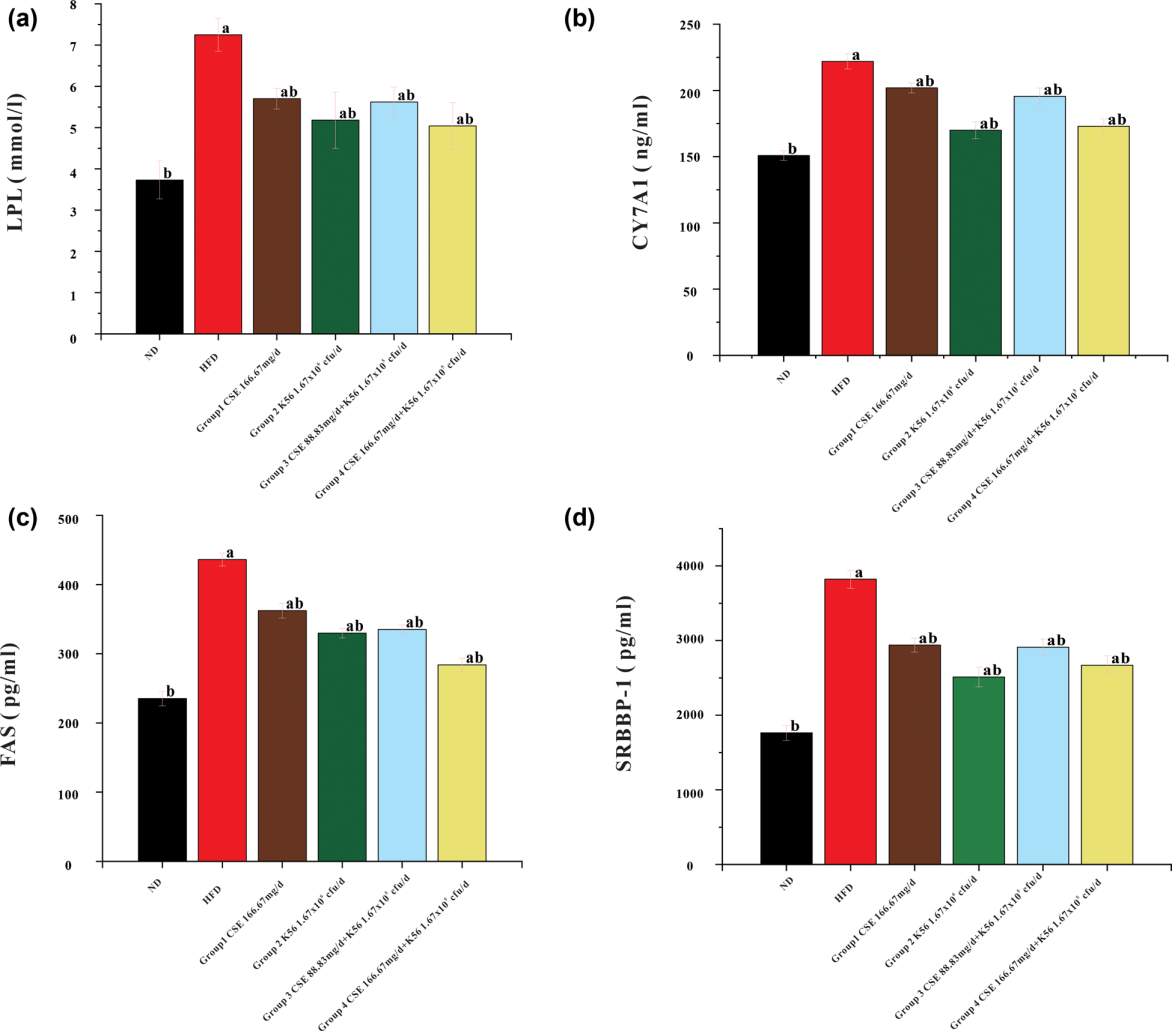
Results of glycolipid metabolism‐related indicators in liver tissue: (a) LPL (lipoprotein lipase); (b) FAS (fatty acid synthase); (c) CYP7A1 (cholesterol 7α‐hydroxylase); and (d) SREBP‐1c (sterol‐regulatory element‐binding transcription factor‐1c). Data are means ± SD (*n* = 12); a: *p* < 0.05, compared with ND, and b: *p* < 0.05, compared with HFD.

## 4 DISCUSSION

Our results showed that under the same feed intake, all interventions could effectively control weight gain, reduce the accumulation of body fat, improve dyslipidemia, reduce FBG levels, improve the blood glucose regulation ability, increase the voluntary exercise, and improve the energy metabolism of obese mice. Although the CSE + K56 intervention contained lower doses of each component than the CSE and K56 alone interventions, it could still effectively control body weight, reduce the fat content, regulate dyslipidemia, and improve the energy metabolism of obese mice. Moreover, these parameters showed no statistical differences between the combined and K56 and CSE alone interventions. However, the CSE + K56 intervention lowered the blood glucose levels faster than the CSE intervention. Compared with the K56 intervention, the CSE + K56 intervention showed similar effects on the lowering of FBG, but a greater ability to regulate blood glucose. The CSE + K56 intervention had higher RER values, more physical activity, and higher EE values than the CSE and K56 alone interventions. These results suggested that the CSE + K56 intervention has a synergistic effect on controlling body weight, reducing fat content, improving dyslipidemia, regulating blood glucose level, and improving energy metabolism.

Chronic low‐grade inflammatory state is a major factor in the progression of chronic metabolic diseases (Balistreri et al., 2010; Hotamisligil, 2006). Obesity increases the endoplasmic reticulum stress and activates the unfolded protein response, which in turn triggers NF‐κB, JNK, and other pathways and increases oxidative stress, thereby upregulating the inflammatory cytokines (Saltiel & Olefsky, 2017). Simultaneously, obesity causes adipocyte hypertrophy in the white adipose tissue and increases FFAs. Furthermore, the changes in adipokines induce inflammatory responses and affect glucose and lipid metabolism (Balistreri et al., 2010; Jiang et al., 2019; Saltiel & Olefsky, 2017; Vorotnikov et al., 2019). Therefore, improving the inflammatory state of obesity and improving the abnormal glucose and lipid metabolism may be the key to delaying the progression of chronic diseases.

Studies have shown that CSE can reduce the content of FFA in blood by regulating the metabolism pathway of adiponectin–adenosine monophosphate–activated protein kinase–acetyl‐CoA carboxylase–malonyl‐CoA–free fatty acid lipid (Jianmin et al., 2017) and increase the content and activity of antioxidant enzymes for reducing the content of serum free radicals (Wang et al., 2012; Xu et al., 2002). In addition, CSE can regulate neuroendocrine activity in the brain, reduce the synthesis of LEP in obese individuals, and control energy balance (Kim et al., 2004, 2007). *L. paracasei* can improve the state of intestinal flora disorder, reduce the levels of bacterial endotoxin lipopolysaccharide (LPS), trimethylamine‐*N*‐oxide (TMAO), and increase the content of short‐chain fatty acids, thereby improving the inflammatory state, regulating glucose and lipid metabolism (Zhao et al., 2021). In obese individuals, reducing fatty acid content, free radical content, LEP level, increasing antioxidant enzyme activity, and improving the state of intestinal flora disorder all help reduce inflammatory factor levels (Bastard et al., 2006; Bouter et al., 2010; Uysal et al., 1997). In this study, it was found that the use of CSE and K56 alone or combined intervention had the effect of reducing serum LEP and ADP levels and reducing serum IL‐1β and TNF‐α.

IL‐1β promotes the release of inflammatory cytokines by mediating islet β‐cell apoptosis or activating the NF‐κB pathway, thereby inducing insulin resistance and the development of type 2 diabetes (Bastard et al., 2006; Petrasek et al., 2012). TNF‐α promotes FFA release through the hormone‐sensitive lipase pathway. Subsequently, FFA can increase the macrophage secretion of TNF‐α through the TLR4/NF‐κB pathway, resulting in a vicious circle of mutual promotion of TNF‐α and FFA. In addition, TNF‐α can participate in the apoptosis and atrophy of brown adipocytes induced by insulin resistance and promote the development of obesity with hyperinsulinemia (Bastard et al., 2006; Bouter et al., 2010; Uysal et al., 1997). In this study, it was also found that after the intervention of the test substance, the RER of obese mice increased and the proportion of glucose in the energy metabolism substrate was increased, which may be related to reducing IL‐1β and TNF‐α levels, reducing insulin resistance and regulating the body's ability to metabolize glucose (Han et al., 2019; Shuwen, 2018).

Studies have shown that mice on a long‐term high‐fat diet show a certain state of depression under a high‐energy load, which reduces the activity of obese mice (Shuwen, 2018; Xiaofeng, 2019; Yiyuan et al., 2013). In this experiment, it was found that the amount of exercise in the HFD group decreased. After the intervention of the test substance, the amount of exercise and energy metabolism in obese mice increased, which may be related to the reduction of serum LEP and ADP levels and the improvement of inflammatory state (Zhang et al., 2015). The test substance intervention improves the inflammatory state of obese mice, regulates the metabolic state of glucose and lipids, improves the depressive state of mice due to hyperglycemia and hyperlipidemia (Yiyuan et al., 2013), and increases the amount of exercise and energy metabolism in obese mice. At the same time, the increase in exercise and the improvement of the inflammatory state can enhance the LEP signaling pathway to regulate POMC neurons in the hypothalamus and regulate the feeding behavior of mice to maintain energy homeostasis (Zhang et al., 2015). Through the results of food intake in this study, it was also found that the total feed intake of the animals in the test substance intervention group was lower than that in the HFD group. The combined intervention of CSE + K56 reduced the level of inflammation better than that of CSE and K56 alone, suggesting that CSE and K56 may play a synergistic role. The composition may regulate the level of serum inflammatory factors, reduce the production of free fatty acids, relieve insulin resistance, and increase energy metabolism through multiple pathways, thereby achieving the effects of controlling body weight and improving blood glucose regulation function.

Endoplasmic reticulum stress is exacerbated by chronic low‐grade inflammatory states (Xue et al., 2005; Zhang & Kaufman, 2008). Hepatocytes are rich in ER content and are responsible for the synthesis of proteins, cholesterol, bile acids, and phospholipids (Zhou & Liu, 2014). Perturbation of ER homeostasis contributes to hepatic steatosis, inflammation, and insulin resistance in the liver (Hotamisligil, 2010). SREBP is a master regulator of hepatic lipid homeostasis, and SREBP‐1c controls fatty acid and triglyceride biosynthesis (Amemiya‐Kudo et al., 2002). The aggravated endoplasmic reticulum stress can promote SREBP‐1c overexpression by regulating the PERK–SREBP‐1–FAS signaling pathway (Wang et al., 2021), thereby overexpressing the FAS protein and promoting adipogenesis and fat accumulation in non‐adipose tissues (Fan‐yong et al., 2007; Notarnicola et al., 2012). Our results showed that either CSE and K56 intervention alone or combined intervention reduced the levels of FAS and SREBP‐1c in obese mice. It is speculated that the weight reduction of the epididymis and perirenal tissue of mice in the intervention groups was caused by the reduction in the inflammation and stress of the endoplasmic reticulum. Regulating the PERK–SREBP‐1c–FAS signaling pathway reduces the content of SREBP‐1 and FAS in the liver tissue, thereby reducing the synthesis and accumulation of fat. The CSE + K56 intervention had a stronger effect on reducing the content of SREBP‐1c and FAS than the CSE and K56 alone interventions. These findings suggested that the combined intervention might be more beneficial than the alone interventions in reducing the content of lipid synthesis‐related enzymes owing to its greater improvement of the inflammatory state. Therefore, the combined intervention may be more beneficial than the CSE and K56 alone interventions in delaying the development of chronic diseases.

## 5 CONCLUSIONS

The interventions of CSE, K56, and CSE + K56 controlled body weight and blood glucose levels and reduced lipids levels. The CSE + K56 intervention displayed synergistic effects on the body weight and fat reduction, dyslipidemia improvement, and blood glucose‐level regulation of obese mice. In addition, the CSE + K56 intervention improved the chronic inflammatory state, reduced the content of lipid synthesis‐related enzymes, and increased energy metabolism more than the CSE and K56 alone interventions. Its advantages may be due to the multichannel regulation, which has synergistic effects and is more conducive to delaying the progression of chronic diseases.

## AUTHOR CONTRIBUTIONS

**Wei Zhang**: Data curation; Formal analysis; Investigation; Methodology; Software; Visualization; Writing – original draft. **Xiuzhen Jia**: Data curation; Methodology; Project administration; Resources; Supervision; Writing – review & editing. **Yuhan Xu**: Investigation; Methodology; Software; Validation. **Qiaoling Xie**: Investigation; Methodology; Supervision; Validation. **Meizhen Zhu**: Formal analysis; Investigation; Software; Visualization. **Zifu Zhao**: Conceptualization; Funding acquisition; Project administration; Resources; Supervision. **Jingyu Hao**: Funding acquisition; Project administration; Supervision; Validation. **Haoqiu Li**: Funding acquisition; Project administration; Resources; Supervision. **Jinrui Du**: Formal analysis; Project administration; Supervision. **Yan Liu**: Data curation; Project administration; Supervision. **Wei‐Hsien Liu**: Conceptualization; Funding acquisition; Validation. **Xia Ma**: Data curation; Project administration; Supervision. **Weilian Hung**: Data curation; Validation. **Haotian Feng**: Conceptualization; Formal analysis; Funding acquisition; Project administration Resources; Validation; Writing – review & editing. **Hongwei Li**: Conceptualization; Data curation; Formal analysis; Investigation; Project administration; Resources; Supervision; Validation; Writing – review & editing.

## CONFLICTS OF INTEREST

The authors declare that they have no conflicts of interest.

## ETHICS STATEMENT

All experimental procedures were performed according to the guidelines of the Institutional Animal Care and Use Committee of the Laboratory Animal Center of Xiamen University and followed the International Association of Veterinary Editors guidelines for the Care and Use of Laboratory Animal. The animal use protocol listed below has been reviewed and approved by the Animal Ethical and Welfare Committee of the Laboratory Animal Center of Xiamen University, Approval No. XMULAC20200185.

## Data Availability

All data generated or analyzed during the current study are available from the corresponding author on reasonable request.
